# New Supramolecular Drug Carriers: The Study of Organogel Conjugated Gold Nanoparticles

**DOI:** 10.3390/molecules26247462

**Published:** 2021-12-09

**Authors:** Joanna Kowalczuk, Andrzej Łapiński, Elżbieta Stolarczyk, Oleg M. Demchuk, Konrad Kubiński, Monika Janeczko, Aleksandra Martyna, Maciej Masłyk, Sylwia Turczyniak-Surdacka

**Affiliations:** 1 Institute of Molecular Physics, Polish Academy of Sciences, 60-179 Poznan, Poland; lapinski@ifmpan.poznan.pl; 2 Łukasiewicz Research Network, Institute of Industrial Chemistry, 01-793 Warsaw, Poland; e.stolarczyk@ifarm.eu; 3 Department of Molecular Biology, Faculty of Science and Health, The John Paul II Catholic University of Lublin, 20-708 Lublin, Poland; kubin@kul.lublin.pl (K.K.); mjanec@wp.pl (M.J.); aleksandra.martyna@kul.lublin.pl (A.M.); maciej.maslyk@kul.pl (M.M.); 4 Biological and Chemical Research Centre, University of Warsaw, 02-089 Warsaw, Poland; sturczyniak@cnbc.uw.edu.pl

**Keywords:** pharmaceutical gels, gold nanoparticles, SEM, Raman spectrometry, diffusiometry, TGA, biological activity

## Abstract

An aqueous solution of sodium citrate stabilized gold nanoparticles (AuNP) in the presence of *N-*lauroyl-L-alanine (C_12_ALA) forms a stable gel. The structure of the gel and the distribution profile of AuNP in it were analyzed. Will nanoparticles separated from each other with sodium citrate behave in the same way in solution and trapped in the gel matrix? Will the spatial limitation of solvent molecules aggregate nanoparticles and destroy their homogeneity? These questions are very important from the point of view of the use of gold nanoparticles, trapped in the gel structure as carriers of drugs in the slow-release process. The lack of homogeneity of this distribution will have a major impact on the rate of release of the appropriate amount of therapeutic drug from the matrix. In this work, we attempt to answer these questions. The performed biological assays revealed that both C_12_ALA and C_12_ALA-AuNP show an excellent level of biological neutrality. They might be used as a transporting medium for a drug delivery without affecting the drug’s activity.

## 1. Introduction

Nanoparticles (NP), the diameter of which is in the range from 1 to 100 nm, are materials that have revolutionized many industries in the last decade [1,2]. Nanotechnology has become a new branch of science that deals with the construction of nanoparticles, and its achievements are also used in pharmacy and medicine [3]. In molecular diagnostics [4], the use of nanoparticles enables detection of cancerous tumors at their early stages of development and faster implementation of therapy. Nanoparticle-based cancer therapy is also effective [5,6,7]. The increase of bioavailability and accumulation of therapeutic compounds, carried by nanoparticles, in the area of tumors significantly improves the prognosis in treatment [7,8]. It is extremely important to search for new compounds that will have potential in medical applications. We need effective compounds that would deliver the active ingredient of the drug to a well-defined place in the human body. However, the transport of such a medicine must be controlled.

Gels are very widely used in medicine for many reasons: these materials are safe, stable, easy to prepare and easily applicable [9,10]. Gels are (at least) a two-phase system in which these phases interpenetrate without formation of new chemical bonds. A rigid gel matrix, made of gelator particles, is only a “skeleton” that maintains the liquid phase in its volume. Increasing the temperature very often starts the processes of gel breakdown, and the liquid phase is slowly released from the rigid matrix. This is characteristic of gels based on cellulose derivatives widely used in pharmacy. For drugs, controlling the rate of active agent administration is a very important factor. We are looking for a gel of which the liquid phase is a solution of nanoparticles, which will be the carrier of an active molecule in the drug, and which, when administered to the patient, will disintegrate and release the therapeutic agent in a controlled and safe manner.

To create the gel, we used the *N-*lauroyl-L-alanine gelator (C_12_ALA), which was obtained by condensation of the two nontoxic natural compounds: natural alanine and fatty lauric acid in conditions we had reported recently [11] and had not yet been used as a gelator for drug delivery. The unusual ability of this gelator to gel organic oils had already been assessed [12,13]. However, not all drugs dissolve in oils (e.g., the popular antibiotic tetracycline, which is polar) and have to be administered as an aqueous solution. That is why, in this work, we present the results of studies on a hydrogel, formed by C_12_ALA and containing stabilized AuNP, the potential drug carriers.

## 2. Sample Preparation

**Figure 1:**
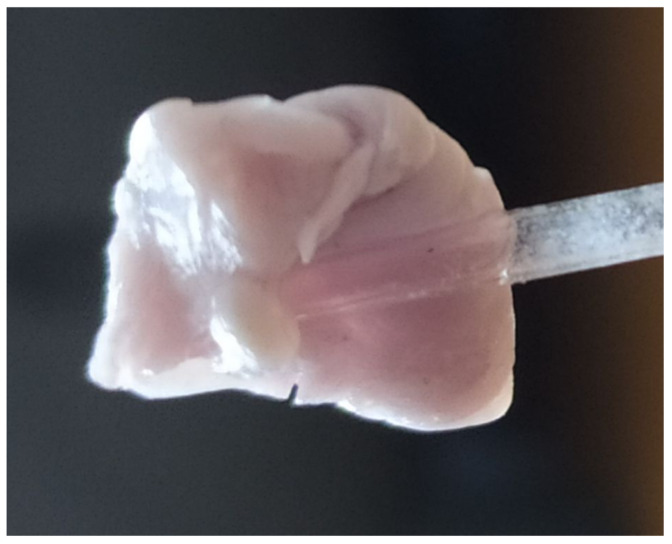
C_12_ALA-AuNP.

The C_12_ALA-AuNP-enriched hydrogel samples were made as follows: to the solution of gold nanoparticles (AuNP) stabilized with sodium citrate, prepared as presented recently [14], at a concentration of 50 mg/L, 6 wt % of C_12_ALA was added. The content of gold nanoparticles in the gel was 0.005%. The mixture was kept at 4 °C for 24 h. Then, the mixture was heated in a stream of warm (~60 °C) air until the gelator aggregates had completely dissolved. The clear liquid was rapidly cooled with a stream of cold water, which led to gelation of the mixture. A stable hydrogel enriched with AuNP was obtained (C_12_ALA-AuNP) as a pink semisolid (see Figure 1).

## 3. Methods

The structure of the rigid matrix of the resulting gel was examined by means of the nuclear magnetic resonance diffusionometry. The relation between the diffusion coefficient of the aqueous fraction in gel and the duration of the diffusion measurement was tested at a room temperature (25 °C) and fever temperature (37 °C). The time-dependent effective diffusion coefficient *D_eff_(*Δ*)* measurements of water molecules in the C_12_ALA organogels network were performed with Bruker Avance 500 MHz (11.75 T) spectrometer equipped with magnetic field gradients using pulsedgradient spin-echo (PGSE) pulse sequence proposed by Stejskal and Tanner [15]. In this method, the measured relation between the echo signal and the magnetic field gradient is described by the formula $A\left(g,\;∆\right)=A\left(0\right)exp\left[-\gamma^{2}g^{2}\delta^{2}D_{eff}\left(∆\right)\left(∆-\frac{\delta }{3}\right)\right]$ where $b_{i}=\gamma^{2}g^{2}\delta^{2}\left(∆-\frac{\delta }{3}\right)$, and A(g, Δ) and A(0) are the echo signal intensities measured at echo time TE with and without the field gradient pulse strength g, respectively. Δ is the gradient pulse interval in Stejskal and Tanner pulse sequence called a diffusion time, δ is the duration of its pulses and γ is the magnetogyric ratio of the proton. As a result of the diffusion experiment, we obtain the dependence of the signal amplitude on *b*_i_. The diffusion time Δ was chosen in the range of 10 to 500 ms with the duration of the gradient δ = 2 ms in all experiments. The diffusion coefficient *D_eff_(*Δ*)* is obtained by fitting the A(g, Δ) to experimental data [16].

Thermal properties and kinetics of obtained hydrogel were examined by TGA thermogravimetry $A\to^{k}B+∆H$, where A is a material which undergoes thermal conversion at a rate of k, at a given temperature, changes into material B, at the same time emitting heat of transformation ΔH. The degree of transformation α in the heating process depends on the number of chemical processes *n* taking place in the material as a function of temperature increase $\frac{d\alpha }{dt}=k{\left(1-\alpha \right)}^{n}$ and $k=Zexp\left(\frac{E_{a}}{RT}\right)$, where Z is a pre-exponential factor (1/seconds), E_a_ is the activation energy (J/mol) and R is a gas constant equal 8.314 J/mol*K. The measurements were carried out on a Perkin Elmer TGA 8000 thermogravimetric analyzer. The TGA method uses three different heating rates, 1, 5 and 10 °C. A kinetic analysis of the gel disintegration process was performed using the dependence of mass loss with increasing temperature according to the Ozawa–Flynn–Wall (OFW) theory. Three temperature dependencies of weight loss percentage were obtained. The total decomposition of the hydrogel was recorded at a temperature of about 300 °C [17,18].

The microscale homogenous distribution of gold nanoparticles (AuNP) in the hydrogel C_12_ALA-AuNP was found by Raman spectroscopy. Raman scattering spectra were recorded on a HORIBA Jobin Yvon LabRAM HR800 spectrometer at room temperature, exciting the sample of C_12_ALA-AuNP with a He-Ne laser beam at 633 nm [19].

In order to visualize the obtained C_12_ALA-Gold gel structures and the distribution of gold nanoparticles inside of it, the scanning electron microscopy (SEM) images were made [20].

For antimicrobial activity, the microbial strains *Staphylococcus aureus* (ATCC 6538), *Escherichia coli* (ATCC 8739), *Klebsiella pneumoniae* (PCM1), *Pseudomonas aeruginosa* (PCM 2562) and yeast *Candida albicans* (ATCC 10,231) were obtained from the Department of Molecular Biology, of The John Paul II Catholic University of Lublin, Poland. The MICs were determined using a microbroth dilution methods [21,22]. The bacterial strains were inoculated in Mueller Hinton Broth medium (Biocorp, Warsaw, Poland) and *C. albicans* was inoculated in Sabouraud Dextrose liquid medium (Biocorp, Warsaw, Poland) and incubated at 37 °C and at 30 °C, respectively, with vigorous shaking (200 rpm) for 24 h. Bacterial cell suspensions at initial inoculums of 5 × 10^5^ in Mueller–Hinton liquid medium and adequate yeast suspensions at initial inoculums of 3 × 10^3^ cfu/mL in RPMI-1640 medium (with 1-glutamine and phenol red, without bicarbonate) (Sigma-Aldrich, Saint Louis, MO, USA) buffered with 0.165 M 3-(N-morpholino)propane sulfonic acid (MOPS) (Sigma-Aldrich, Saint Louis, MO, USA) were exposed to the examined compound at relevant concentrations (range 0.14–8.8 mM) for 24 h at 37 °C for the bacteria or for 48 h at 37 °C for the fungi. The MIC was the lowest concentration of the compounds that inhibited the visible growth of the microorganism. The experiments were performed in triplicate.

The stock solutions of the tested compounds were prepared in DMSO in such a way that the final concentration of DMSO does not exceed 0.01%. For antiproliferative activity assay, The Detroit 562 and HeLa cell lines were obtained from ATCC. Cells were cultured in DMEM (Dulbecco’s Modified Eagle Medium, high glucose) + GlutaMAX supplemented with penicillin (100 U/mL), streptomycin (100 U/mL) and 10% heat-inactivated FBS. Cells were maintained in a humidified atmosphere at 37 °C and 5% CO_2_ and passaged twice before performing an experiment. For antiproliferative activity, assay cells of each cell line were seeded in 96-well microplates (Wuxi Nest Biotechnology, Wuxi, Jiangsu, China) at a density of 2.5 × 104 cells/mL in 100 μL DMEM + GlutaMAX supplemented with 10% heat-inactivated FBS. After 24 h of cell attachment, plates were washed with 100 μL/well with Dulbecco’s phosphate-buffered saline (DPBS) and the cells were treated with increasing concentrations of C_12_ALA and C_12_ALA-AuNP compound (1–100 µM final concentration) prepared in fresh FBS-free medium for 48 h. Each concentration was tested in triplicate. Sets for both cell lines included wells containing 0.01% DMSO as a negative control. Antiproliferative activity of compounds was assessed using MTT assay, as described below.

Following 48 h of compound exposure, the control medium or the test exposures medium was removed, the cells were rinsed with DPBS and 100 μL of fresh medium (without FBS or antibiotics) was added to each well. Next, 10 μL of MTT (5 mg/mL) prepared in DPBS were added to each well and the plates were incubated for 3 h at 37 °C in a 5% CO_2_ humidified incubator. After the incubation period, the medium was discarded, the cells were washed with 100 μL of DPBS and 100 μL of DMSO was added to each well to extract the dye. The plate was shaken for 10 min and the absorbance was measured at 570 nm. Viability was calculated as the ratio of the mean of OD obtained for each condition to the control condition.

The hemolytic activity of the C_12_ALA and C_12_ALA-AuNP was determined on human red blood cells. Human erythrocytes were harvested by centrifugation for 10 min at 2000 rpm and 20 °C, and washed three times in phosphate-buffered saline (PBS). To the pellet, PBS was added to yield a 10% (*v*/*v*) erythrocytes/PBS suspension. The 10% suspension of erythrocytes was then further diluted with PBS at a 1:10 ratio. Then, 450 μL of the final diluted erythrocytes were added to 50 μL of PBS having a previously determined concentration gradient (MIC to 5×MIC) of the test compounds in microcentrifuge tubes. Total hemolysis was attained in 1% Triton X-100. The tubes were incubated for 1 h at 37 °C and then centrifuged for 10 min at 2000 rpm at room temperature. From the supernatant fluid, 150 μL were transferred to a flat-bottomed microtiter plate, and the absorbance was measured spectrophotometrically at 450 nm. The hemolysis percentage was calculated by the following equation: % hemolysis = (A_450_ of test compound treated sample − A_450_ of buffer treated sample/ A_450_ of 1% Triton X-100 treated sample − A_450_ of buffer treated sample) × 100%.

## 4. Results

**Figure 2:**
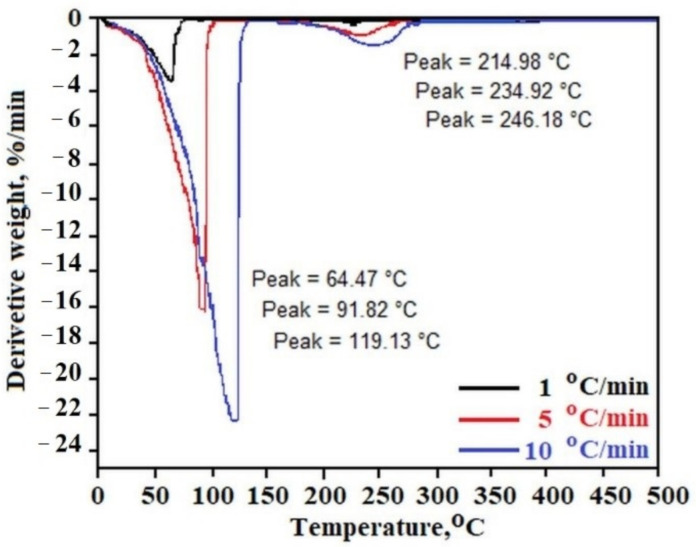
Temperature dependence of the derivative of weight loss of C_12_ALA-AuNP measured in a function of heating rates by TGA.

Figure 2 presents the temperature dependence of the derivative (weight loss on time) of C_12_ALA-AuNP measured with heating rates of 1 °C/min, 5 °C/min and 10 °C/min for black, red and blue lines, respectively. In all experiments, two characteristic peaks can be distinguished. The first peak, obtained in the range of 64 to 120 °C, is connected with the solvent evaporation during the heating process. The second one, observed between 220 and 250 °C, is characteristic of gel rigid matrix decomposition. The increase of the sample heating rate in the TGA experiment shows the increase of the decomposition rate of both gel components (solvent and rigid matrix). For 1 °C/min of heating rate, the rates of weigh loss of liquid and solid part of gel are equal, 3.356 %/min and 0.157 %/min, respectively, whereas these parameters increased to 22.040 %/min and 1.311 %/min for 10 °C/min of TGA heating rate.

**Figure 3:**
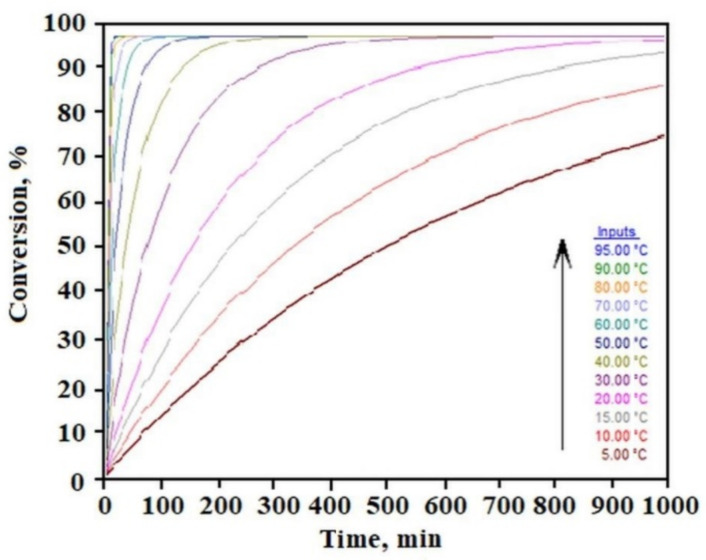
Time dependence of sample conversion as a function of temperature obtained by OFW theory.

The results obtained directly from the measurement of mass loss with increasing temperature in the TGA experiment can be analyzed according to the OFW theory showing the kinetics of the sample disintegration process. The temperature area in which the solvent evaporation from the sample takes place was analyzed. The constant *k* was determined for each decomposition rate for 10, 15, and 20 percent weight loss for one type of reaction *n –* solvent evaporation. Figure 3 shows the time dependence of the sample decomposition (conversion) as a function of temperature. This analysis allows to establish safe storage conditions for the sample. By setting an acceptable limit for the weight loss of the liquid component in the gel in separate tests, we can determine the gel’s residence time under different temperatures based on this relation. For example, for the limit of 10% liquid phase loss, we can see that C_12_ALA-AuNP can stay at a temperature of 10 °C for about 70 min, whereas increasing the temperature to 90 °C shortens this time to a few minutes.

**Figure 4:**
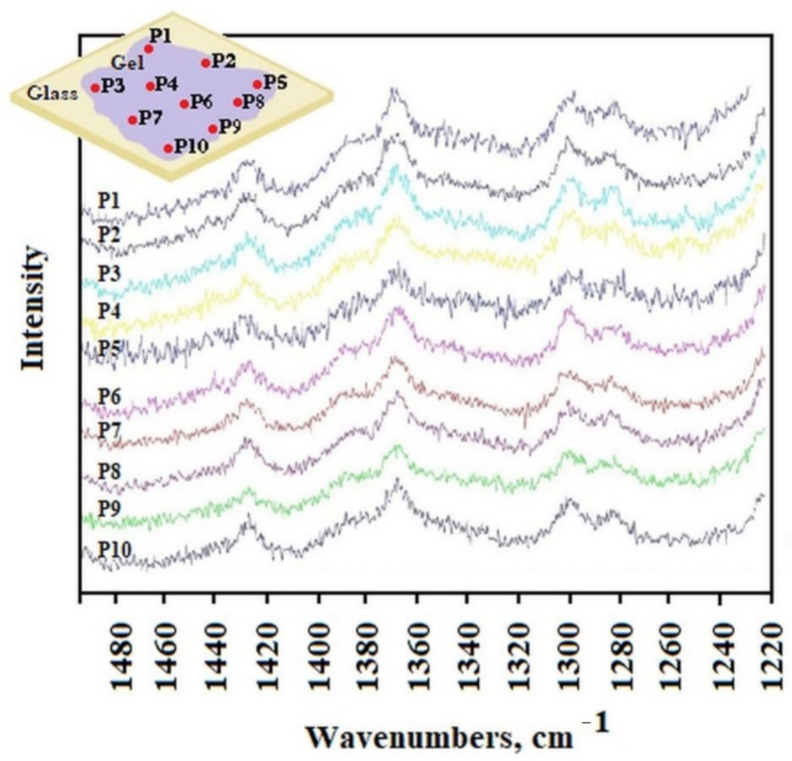
Raman spectra as a function of position on the surface (from P1 to P10) of the C_12_ALA-AuNP sample.

The distribution of AuNP in the gel was studied by Raman spectroscopy. The He-Ne laser beam at 633 nm was positioned to obtain the appropriate spectra from different separated locations P1 to P10 of the sample placed on a glass surface. Figure 4 shows the Raman spectra obtained from different laser locations on the surface (from P1 to P10). The surface of the sample on the laboratory glass was about 1 cm^2^, at each subsequent laser position, its light fell on a small part of the sample with the spot size of 1 μm. All spectra were very similar and show characteristic peaks at the same wavelengths. This evidenced a high degree of sample homogeneity. However, this study did not show whether the AuNP were in each of the observed places, because locating the gold nanoparticle characteristic peak proved impossible without being able to compare it with the appropriate spectrum.

**Figure 5:**
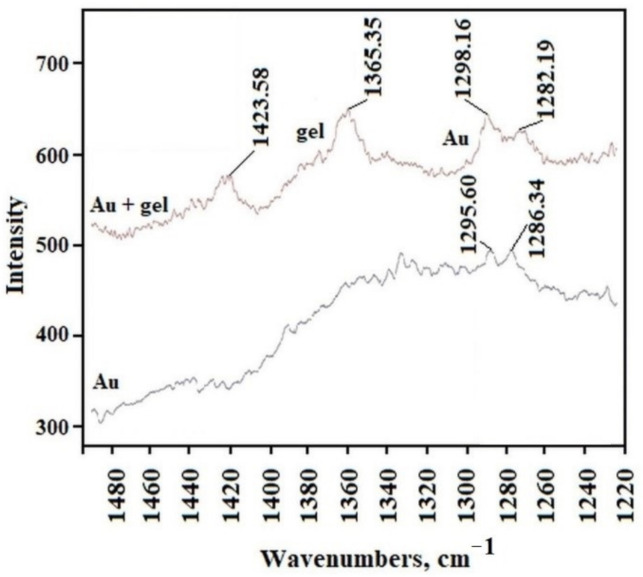
The comparison of Raman spectra for C_12_ALA-AuNP and gold nanoparticles.

To record the Raman spectrum of AuNP, we separated precipitated gold agglomerate from the liquid phase of overheated C_12_ALA-AuNP. The analysis of the Raman spectra indicates that the average value of ratio of intensity of the two peaks characteristic for C_12_ALA and gold nanoparticles obtained in different places on the surfaces equals 1.48, and in most places the deviation from this value does not exceed 10%. We can conclude that the distribution of gold nanoparticles is homogenous in the C_12_ALA-AuNP.The comparison of C_12_ALA-AuNP and AuNP Raman spectra is shown in Figure 5.

The gold nanoparticles and the gel sample characteristic peaks can be distinguished on these spectra. The peaks obtained for the wavelength of about 1295.60 cm^−1^ and 1286.34 cm^−1^ in the gold sample are visible in every position on gel surface; however, other peaks recorded in the gels are not visible in the gold sample. The shift of these two peaks in the gel compared to those in Au, from 1295.60 cm^−1^ and 1286.34 cm^−1^ to 1298.16 cm^−1^ and 1282.19 cm^−1^, respectively, is due to the interaction between the solvent and gelator molecules in the gel structure, which is typical for molecular gels [23]. Based on this observation, we can conclude that the distribution of gold nanoparticles in the C_12_ALA-AuNP is quite homogenous on the observed scale.

**Figure 6:**
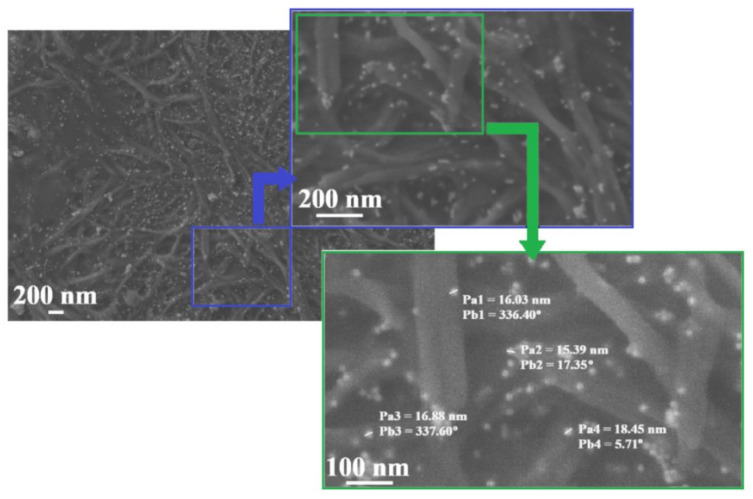
The SEM images of C_12_ALA-AuNP surface. The image fragment with a resolution of 100 nm shows the size (Pa1–Pa4) and position (Pb1–Pb4) of gold nanoparticles in the gel matrix.

The conclusion made was confirmed by SEM tests of the investigated C_12_ALA-AuNP sample. The SEM images of the C_12_ALA-AuNP surface sample with different resolution are shown in Figure 6.

As we can see, the structure of a rigid gel matrix consists of interpenetrating fibers of a fairly uniform thickness. AuNP are scattered on fibers. In the image showing the largest part of the sample, we can see that the distribution of AuNP is almost homogenous and there are not too many aggregates of nanoparticles. At different positions in the sample (from Pb4 = 5.71° to Pb3 = 337.60°), gold nanoparticles are separated from each other and visible in the photo as brighter dots with the dimensions from Pa2 = 15.39 nm to Pa4 = 18.45 nm.

**Figure 7:**
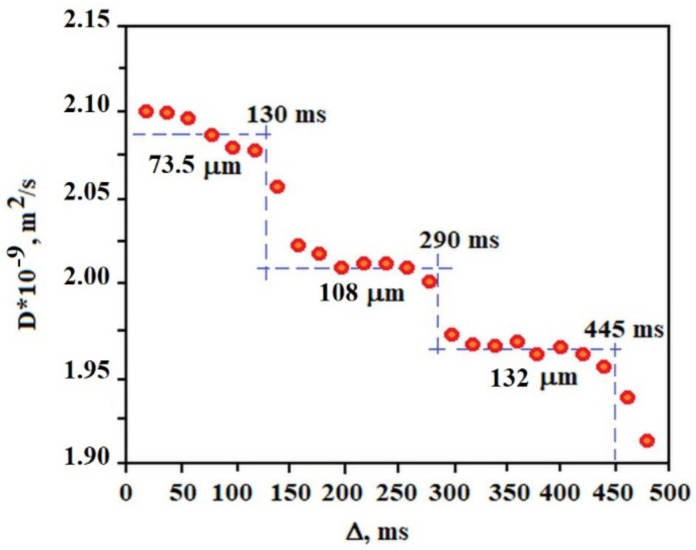
The diffusion time dependence of diffusion coefficient *D_eff_(*Δ*)* of solvent molecules in C_12_ALA-AuNP.

Figure 7 shows the dependence of the diffusion coefficient of the solvent molecules (average diffusion value of water molecules, AuNP and sodium citrate) in the gel on the diffusion time measured at 25 °C. Each plate obtained in a given range of diffusion times allows the calculation of the approximate diameter of the pores in which diffusion occurs. This result indicates that the gel structure is ordered in terms of the pore size in which the solvent molecules diffuse. Of course, in the gel sample a very large pore size distribution exists but several of them dominate the sample, 73.5 μm, 108 μm, and 132 μm in the range of Δ from 0 to 130 ms, 130 ms to 290 ms, 290 ms to 445 ms, respectively. Such a result (“stepped”) is characteristic of the so-called restricted diffusion, which disappears when the internal order of the gel in terms of pore size is destroyed by the temperature or other external factors. In the case of a C_12_ALA-AuNP gel, increasing the temperature to 37 °C resulted in an increase in the diffusion coefficient and made it independent of the diffusion time in relation to the lower temperature. This proves the high dynamics of the gel as a function of temperature and the activation of faster transport of the liquid phase from the gel at higher temperatures, which is desirable from a medical point of view because this indicates the possibility of a temperature-controlled release of the drug from the gel.

The compounds were tested against selected microorganisms, human cell lines and human erythrocytes (Table 1).

Antimicrobial activity was examined towards four bacteria and one fungal strains. Both C_12_ALA and C_12_ALA-AuNP show low activity by inhibiting only the growth of *S. aureus* at high concentrations of 1.1 mM and 1.8 mM, respectively, while *C. albicans* is affected only by C_12_ALA-AuNP at the concentration of 1.8 mM. Since systemic antimicrobial drugs show MIC values at the nanomolar to low micromolar range, the tested chemicals exhibit very poor antimicrobial activity [24,25,26].

Next, the anticancer activity of C_12_ALA and C_12_ALA-AuNP was examined against human HeLa and Detroit 562 cell lines. The activity was also low, the compounds at the concentration of 100 µM inhibit the proliferation of cancer cell lines, namely HeLa and Detroit 562 only by 17.4% and 4.7–18.8 %, respectively. It must be noted that anticancer compounds are substances with IC_50_ values at the nanomolar-to-low-micromolar level [27,28].

Finally, the human erythrocytes were used to examine the hemolytic activity of the tested compounds. The results show that up to the concentration of 1 mM and 0.02 mM for C_12_ALA and C_12_ALA-AuNP, respectively, the compounds do not cause any hemolysis of the blood cells. However, at the concentration of 5 mM and 0.1 mM, 22.2% and 10.9% of the blood cells were lysed by the C_12_ALA and C_12_ALA-AuNP, respectively. Taking the performed biological assays into consideration, both C_12_ALA and C_12_ALA-AuNP do not show or show very low biological activity.

## 5. Conclusions

The research allows us to answer the questions posed in the abstract of the work. Will gold nanoparticles separated from each other with sodium citrate behave in the same way in solution and trapped in the gel matrix? The answer is YES. The Raman spectroscopy and SEM images clearly show the separation and homogenous distribution of AuNP in C_12_ALA gel. Will the spatial limitation of solution molecules aggregate nanoparticles and destroy their homogeneity? The answer is NO. The intensity of the peaks characteristic of gold nanoparticles in the analysis of Raman spectra clearly indicate a homogenous distribution of gold nanoparticles in the sample, which is also important from the point of view of the applicability of the C_12_ALA-AuNP; the investigated material was stable below 35 °C. What is more, the biological assays revealed that both C_12_ALA and C_12_ALA-AuNP exhibit very low or no activity against microorganisms, human cancer cell lines, and human erythrocytes; thus, they can successfully be used as a transporting medium of a drug to the place in the human body without affecting the drug’s action.

We can say with high probability that, in the search for a gel enriched with nanoparticles that will retain its structure and homogeneity of the distribution of nanoparticles in a solution trapped in the gel matrix under certain temperature conditions, we are on the right track.

## Figures and Tables

**Table 1:** Biological activity of C_12_ALA and C_12_ALA-AuNP*.

Antimicrobial Activity, Minimal Inhibitory Concentration (MIC)					
Strain		C_12_ALA		C_12_ALA-AuNP	
*E. coli*		-		-	
*P. aeruginosa*		-		-	
*S. aureus*		1.1 mM		1.8 mM	
*K. pneumoniae*		-		-	
*C. albicans*		-		1.8 mM	
**Anticancer Activity, Percent of Living Cells at 100 µM (C_12_ALA or C_12_ALA-AuNP) after 48 h**					
**Cell line**		**C_12_ALA**		**C_12_ALA-AuNP**	
HeLa		-		82.63%	
Detroit 562		95.3%		81.21%	
**Hemolytic Activity, Percent of Hemolysis**					
**C_12_ALA concentration**			**C_12_ALA-AuNP concentration**		
1 mM	0%		0.02 mM		0%
5 mM	22.2%		0.1 mM		10.9%

- no activity; *the concentration of **C_12_ALA-AuNP** is given as concentration of **C_12_ALA** component, while the total concentration of gold is 10^−3^ time smaller.

## Data Availability

Not applicable.

## Sample Availability

Samples of the compounds are available from the authors.
